# Editorial: Modulation of Macrophage Signaling Pathways During Bacterial Infections

**DOI:** 10.3389/fcimb.2021.689759

**Published:** 2021-06-15

**Authors:** Supriya Shukla, Jaya Telraja, Manisha Yadav, Hridayesh Prakash

**Affiliations:** ^1^ Department of Pathology, Case Western Reserve University and University Hospitals Cleveland Medical Center, Cleveland, OH, United States; ^2^ Department of Internal Medicine, Division of Pulmonary, Critical Care and Sleep Medicine, Wayne State University School of Medicine and Detroit Medical Center, Detroit, MI, United States; ^3^ Dr. B.R. Ambedkar Center for Biomedical Research, University of Delhi, Delhi, India; ^4^ Amity Institute of Virology and Immunology, Amity University, Noida, India

**Keywords:** pattern recognition receptors (PRRs), innate immune response, intracellular pathogens, myeloid cells, host-directed therapy (HDT), iNOS+ M1 macrophage

## Introduction

Macrophages are the specialized cells of an immune system that play both an immunological and a physiological role. In response to environmental clues, macrophages change their plasticity. Based on a classical paradigm, macrophages are classified as M1 or classically activated macrophages and M2 or activated macrophages. The phonotypical plasticity of macrophages enables them to contribute to the pathogenesis as well as homeostasis mechanisms. We have been exploring macrophages in the context of a bacterial infection.

We previously demonstrated that M2 polarization of M1 effector alveolar macrophages during chronic/persistent *Chlamydia pneumonia*, *Mycobacterium tuberculosis*, and *Helicobacter pylori*, pathogens are decisive for infection-induced cancer development in a host. Since chronic infection with these pathogens has been associated with adenocarcinoma, we feel that disruption of macrophage plasticity plays a crucial role in the development of cancer. We argue that the management of M1/M2 imbalance is paramount for minimizing the risk of developing cancer by a chronic and persistent infection. This may be achieved by targeting a major intracellular signaling component such as Sphingolipids, Th2/Th17 responses which promote the M2 phenotype during persistent infection and are potentially involved in the development of cancer.

Several articles in this Research Topic discuss how various pathogens sabotage effector functions of M1 macrophages and evade immune control to establish an infection. This Research Topic is a collection of reports that address the importance of macrophage directed approaches in host-directed therapies (HDT) for the effective management of infectious diseases. These bacterial pathogens include *Chlamydia pneumoniae, Chlamydia trachomatis, Salmonella enterica, Listeria monocytogenes*, and *Mycobacterium Tuberculosis* (*M.tb*), which can cause serious human diseases like acute lower respiratory tract infections, typhoid, and tuberculosis (TB). Among these ailments, TB, one of the oldest known human diseases, is a leading cause of death from a single infectious agent (WHO, 2017). People with an impaired immune function are at a heightened risk of developing active disease, making TB co-infection a leading cause of death among people with HIV. Despite the availability of improved diagnostics and treatment options, intracellular bacterial diseases continue to pose a major public health challenge worldwide. The emergence of drug resistance pathogens, such as methicillin-resistant *Staphylococcus aureus* (MRSA), multi-drug resistant (MDR), and others, further dampens the prospect of eradicating these infectious diseases. A greater understanding of the mechanisms of host-pathogen interactions and rewiring of host signaling pathways during infection will aid in the development of novel HDT and therapeutic targets. The bacterium, on the other hand, manipulates macrophage signaling pathways and interferes with host protective responses to survive and establish infection. More work is needed to fully understand the complexity of intracellular infections and host-pathogen interactions. Identifying host factors that are exploited by pathogens will help develop targets for HDT.

This Research Topic was launched to discuss the latest trends in the field and to discuss possibilities of tweaking an effector arm of immunity for controlling the pathogen burden in host cells. Several leading groups across the globe have contributed to this Research Topic and covered various intriguing aspects of signaling, including host pathogen interaction during acute infection in macrophages.

The work by Yu et al. addresses the impact of glycolysis and oxidative phosphorylation in macrophage differentiation during anti-infection and anti-tumor inflammation. They unraveled the importance of HIF1α-mediated glycolysis, which is important for M1 macrophage differentiation and plays a critical role in anti-bacterial infection. Additionally, they demonstrated that active glycolysis may also help prevent excessive reactive oxygen species (ROS) production when mitochondrial respiration is impaired.

The research article by Arora et al. highlights the influence of *M.tb* Rv1954A protein on the M1 polarization of macrophages. They provide experimental evidence that Ms_Rv1954A fosters infectivity and augments the generation of ROS/reactive nitrogen species and apoptosis in RAW264.7 macrophage cells. Rv1954A imparts protection against oxidative and nitrosative stress and enables the survival of Ms_Rv1954A inside macrophages. Although the authors demonstrated the role of *M.tb* Rv1954A in a *Mycobacterium smegmatis* model, this work warrants further investigation in the context of a *Mycobacterium tuberculosis* infection.

Sharma et al. discovered the novel role of the polymorphic guanine-cytosine-rich sequence (PGRS) domain of *M.tb* Rv0297. Their work reveals the impact of Rv0297-PGRS in modulating calcium homeostasis and the arrest of acidification of the phagolysosomal compartment in macrophages. They explicitly demonstrate that macrophages infected with recombinant *M. smegmatis* (r*M.smeg)* expressing Rv0297 produce nitric oxide and undergo apoptosis which may aids in the dissemination of pathogen in the later stages of infection. Rv0297 was also found to be involved in rescuing the bacterium from oxidative/hypoxic stress, contributing to the survival mechanism of bacteria in the hostile environment of infected macrophages. Their work suggests that Rv0297-encoded PE_PGRS5 may have a role in the calcium-modulated host responses during *M.tb* infection *via* altering macrophage functions.

Garg et al. investigated the functional importance of calcium transporting P2A ATPase, CtpF on the interaction of *M.tb* with macrophages. The authors demonstrated that CtpF enhances calcium effluxes in infected macrophages during the early stages of *M.tb* infection. Downregulation of ctpF expression by conditional knockdown resulted in the perturbation of the host’s calcium levels with a concurrent reduction in mTOR activation in macrophages. Their work demonstrated how *M.tb* engages its metal efflux pumps to exploit autophagy in a host for securing their survival and growth.

The research article by Kim et al. investigated the host immune response against *M. avium* by transcriptome analysis of canine peripheral blood mononuclear cells. The study highlights the temporal regulation of the immune response by *M. avium*. In addition, it reveals that *M. avium*, like *M.tb*, is capable of inducing Th17 immune response and conferring resistance toward apoptosis in infected canine polymorphonuclear leukocytes. This study adds to our understanding as to how *M avium* modulates a host immune response and how we can intervene to afford protection against this pathogen.

The review article by Maphasa et al. discussed the mechanistic aspects of autophagy and its evasion by *M.tb.* Because of the overlap and conserved pathways which orchestrate both autophagy and apoptosis, the authors also focused on the relationship between apoptosis and autophagy during the infection. In view of this, the authors highlighted the therapeutic impact of autophagy inducing compounds (AICs) in the context of *M.tb* infection. The authors also discussed the potential of developing nanoparticles (NPs) based on nanomedicine directed therapies (HDTs) for TB therapy. The introduction of NPs to HDT-TB can allow for targeted drug delivery, therefore minimizing toxic side effects, while increasing the amount of the drugs that reach target cells. Their study also enhanced our understanding of the mode of action of AICs and advised us toward designing a superior treatment strategy against *M.tb* infections in the future.

Another compelling study by Ali et al. reported the novel role of *M.tb* Rv1523 MTase in the methylation of mycobacterial cell envelope lipids and its contribution to virulence and drug resistance. Their findings reveal the hitherto unknown role of Rv1523 encoded MTase in cell wall remodeling and modulation of immune responses. Their data prudently demonstrated that a functional gain of mycolic acid Rv1523 methyltransferase contributes to the virulence and development of resistance to antibiotics in *M. smegmatis*. Thus, mycolic acid methyltransferase may serve as a potential target for the development of novel therapeutics. Moreover, the authors highlight that the Rv1523 MTase is important for maintaining a resilient cell wall structure and also exhibits immune-modulatory properties, which enables the bacteria to persist longer inside the macrophages. The functional role of cell wall MTases in the pathophysiology of *M.tb* makes the Rv1523 mycolic acid MTases enzyme an attractive target for the development of novel drugs against TB.

In a similar study, Lei et al. confirmed that Rv3722c interacts with the host TRAF3 to promote *M.tb* replication in macrophages. A knock-down of the TNF receptor associated factor 3 (TRAF3) attenuated the effect of Rv3722c on the intracellular *M.tb* survival. The authors reveal that Rv3722c interacts with TRAF3 and interrupts its downstream pathways to promote *M.tb* survival in macrophages. The authors also demonstrated that *M.tb* Rv3722c regulates the secretion of cytokine through mitogen-activated protein kinase (MAPK) and nuclear factor kappa light chain enhancer of activated B cells (NF-κB) pathways. In this study, the authors revealed that Rv3722c may inhibit the MAPK pathway by suppressing the phosphorylation of p38 and c-Jun N-terminal kinase (JNK) through TRAF3. These findings facilitate a further understanding of the mechanism of *M.tb* secreted proteins in regulating the host cell immune response and promoting its intracellular survival.

A mini review by Saha et al. discusses the significance of Coronin 1-dependent signaling cascades and the PknG related sequence of events on the pathogenesis of mycobacterial diseases. Coronin 1, being a cortical protein, is transiently recruited to all mycobacteria containing phagosomes, but only pathogenic mycobacteria can retain it on the phagosome to hinder its maturation. The review discusses recent information on these two proteins in the context of macrophage signaling manipulation encompassing diverse mechanisms like calcium-calcineurin signaling, reduced proinflammatory cytokine secretion, cytoskeletal changes, and adaptation in an acidic environment, which ultimately converge toward mycobacterial survival inside the macrophages.

Apart from *M.tb*, *H pylori* is another deadly pathogen that also poses a serious threat and causes not only infection but also cancer. In this context, a compelling study by Xie et al. sheds new light on the mechanisms of the immunopathology of *H. pylori* infection. Their study established a correlation of a notch signaling pathway with an altered mRNA profile in CD4+ Th cells in an *H. pylori* infected patient group. They revealed that increased Notch1 signaling is associated with an enhanced Th1 response during an *H. pylori* infection. The study suggests Notch1 as a possible therapeutic target for the control of an *H. pylori* infection. However, their study could not confirm the influence of Notch1 signaling in Th17 cells which are decisive for the pathogenesis of the disease, and thus need further confirmation for a better understanding of prognosis in *H. pylori*–infected patients.

Leseigneur et al. report an update on the various immune evasion strategies employed by several pathogens in the M1 effector macrophage. Their study was focused on several fascinating subversive tools which are developed by *Listeria monocytogenes, Staphylococcus aureus*, and pathogenic *Yersinia* spp., illustrating diversity and commonality in mechanisms used by microorganisms with different pathogenic lifestyles.

Slonova et al. demonstrated that human recombinant PGLYRP1/Tag-7/PGRP-S (peptidoglycan recognition proteins) potentiates the response of murine (ANA-1 cells) and human macrophages to facultative intracellular pathogen *Listeria monocytogenes*. The authors demonstrated that human PGLYRP1 augments an immunogenic response in mouse macrophages. Human PGLYRP1 also protected mouse macrophages from *L. monocytogenes* infection by enhancing intracellular bacterial killing and promoted immunogenic inflammation. The authors suggest that PGLYRP1/Tag-7/PGRP-S acts as a molecular sensor for an *L. monocytogenes* infection and leads to increased killing through a mechanism(s) that remains to be defined. This is anticipated to be mediated by a scavenger receptor but needs further investigation.

A research article by Liu et al. analyzed the distribution of monocyte subsets in syphilis patients and studied the effect of *Treponema pallidum* (*Tp)* on monocyte functions to explore the pathogenesis of syphilis. *Tp* inhibits the maturation of CD14+/CD16- monocytes into immature phenotype (CD14^+/^CD16^+^ monocytes), promotes secretion of interleukin 1 beta (IL-1β) and increases monocyte migration capacity through the mTOR pathway. Their data revealed that mTOR affects the expression of IL-1β and migration in Tp-exposed THP-1 cells. This study sheds new light on the mechanism by which Tp interacts with monocytes during infection.

A study by Gao et al. reports that macrophage dynamin-related protein 1 (DRP1) promotes the secretion of TNF-α during infection. Their data revealed that DRP1 promotes mitochondrial fission and maintains cellular homeostasis during infection. In this study, the authors confirmed that the depletion of DRP1 decreased mitochondrial fragmentation and Tumor necrosis factor- alpha (TNF-α) production in macrophages upon stimulation with either lipopolysaccharide (LPS) or MRSA infection. Dynamin-related protein 1 (DRP1) was required for the post-transcriptional processing of a TNF-α peptide during infection. The results of the study highlight DRP1 as a key player in the macrophage pro-inflammatory response and suggested its involvement in the post-transcriptional control of TNF-α production.

In their review, Sachdeva and Sundramurthy discuss the various mechanisms by which pathogen containing vacuoles intersect with lysosomal trafficking pathways and maintain their distinctiveness. Their study suggests that different pathogens or pathogen-derived products exhibit a global influence on the integrity of a host lysosomal system. This review integrates recent advancements in how pathogens alter lysosomal pathways and how a host encounters these alterations to restore the protective role of the lysosomes against these pathogens. The review also briefly discusses the heterogeneity in lysosomal targeting by these pathogens and the possible mechanisms and consequences.

A review article by Szulc-Dabrowska et al. summarizes the role of cathepsins in bacteria-macrophage interaction. It describes the important strategies engaged by bacteria to manipulate the cathepsin expression and activity in macrophages. The presence of cathepsins in the endo-lysosomal compartment permits direct interaction with and killing of bacteria and may contribute to the processing of bacterial antigens for effective presentation. The main focus was on specific bacterial species due to their clinical relevance to humans and animal health, i.e., *Mycobaterium, Mycoplasma, Staphylococcus, Salmonella, Shigella, Francisella, Chlamydia, Listeria, Brucella, Helicobacter, Neisseria*, and other genera.

The mini review article by Qian et al. discusses the role of proline-glutamate (PE)/proline-proline-glutamate (PPE) proteins, which are believed to be linked to ESX system function. Furthermore, the authors highlight the importance of PE/PPE proteins in host cell interaction, immune response regulation, and cell fate determination during complex host-pathogen processes. Qian et al. also propose future directions for PE/PPE protein research and rationalize how PE/PPE based diagnostics can be used for global tuberculosis control.

Mishra et al. investigated microRNAs as a therapeutic candidate in HDTs upon bacterial infection. The authors re-analyzed the publicly available transcriptomic dataset of macrophages, infected with different pathogenic bacteria and identified significant genes and microRNAs common to the differential infections. Mishra et al. identified miR-30e-5p, to be upregulated in different bacterial infections which enhances innate immunity to combat bacterial replication by targeting key negative regulators such as *SOCS1* and *SOCS3* of innate immune signaling pathways. The authors proposed miR-30e-5p as one of the potential candidates to be considered for additional clinical validation toward HDTs. Given the significance of miR based therapeutics against cancer and infectious disease, *in vivo* validation of this concept would be advantageous.

The review article by Galli and Saleh outlines recent evidence, highlighting host-bacterial immune-metabolic interactions. Their study emphasized that in response to bacterial sensing; macrophages undergo metabolic adaptations that contribute to the induction of innate immunity signaling and/or macrophage polarization. The intermediates of the glycolysis pathway, the tricarboxylic acid cycle, mitochondrial respiration, amino acid, and lipid metabolism directly interact with and modulate macrophage effectors at the epigenetic, transcriptional, and post-translational levels. Some intracellular bacterial pathogens usurp macrophage metabolic pathways to attenuate anti-bacterial defenses.

## Conclusion and Perspective

The collection of focused research and review articles in this Research Topic provides a herbarium of key findings that sheds new light on various intriguing aspects of infection biology in macrophages. We believe that this Research Topic adds valuable information to understanding to develop HDT, particularly, by exploiting macrophage directed strategies for pathogen control and better clinical outcome of diseases.

## Author Contributions

All authors have contributed to the editing and writing equally. All authors contributed to the article and approved the submitted version.

## Conflict of Interest

The authors declare that the research was conducted in the absence of any commercial or financial relationships that could be construed as a potential conflict of interest.
